# Retinal vessel diameters, flicker‐induced retinal vasodilation and retinal oxygen saturation in high‐ and low‐risk pregnancy

**DOI:** 10.1111/aos.14696

**Published:** 2020-12-16

**Authors:** Mozhgan Sharifizad, Doreen Schmidl, René M. Werkmeister, Harald Zeisler, Reinhard Told, Julia Binder, Lorenz Küssel, Gerhard Garhöfer, Leopold Schmetterer

**Affiliations:** ^1^ Department of Clinical Pharmacology Medical University of Vienna Vienna Austria; ^2^ Center for Medical Physics and Biomedical Engineering Medical University of Vienna Vienna Austria; ^3^ Department of Obstetrics and Gynecology Medical University of Vienna Vienna Austria; ^4^ Department of Ophthalmology Medical University of Vienna Vienna Austria; ^5^ Singapore Eye Research Institute Singapore Singapore; ^6^ School of Chemical and Biomedical Engineering Nanyang Technological University Singapore Singapore; ^7^ SERI‐NTU Advanced Ocular Engineering (STANCE) Singapore Singapore; ^8^ Ophthalmology and Visual Sciences Academic Clinical Program Duke‐NUS Medical School Singapore Singapore; ^9^ Institute of Clinical and Experimental Ophthalmology Basel Switzerland

**Keywords:** biomarkers, flicker stimulation, pregnancy, retinal oximetry, retinal vessels, Clinical Trial Database Number, NCT02340442

## Abstract

**Purpose:**

To compare retinal vascular parameters between high‐risk and low‐risk pregnant women over time during pregnancy.

**Methods:**

In a longitudinal study, we included pregnant women with normal blood pressure and normal body mass index (BMI, group 1), pregnant women with systemic hypertension and/or overweight (group 2) and age‐matched nonpregnant healthy women (group 3). Using the dynamic vessel analyser (DVA) we investigated flicker‐induced vasodilation in retinal arteries (FLA) and veins (FLV), central retinal arterial and vein equivalent (CRAE, CRVE), arterio‐venous ratio (AVR) and retinal arterial and venous oxygen saturation (SartO_2_, SveinO_2_). Study visits were scheduled 2nd trimester (TP 2), 3rd trimester (TP 3) and postpartum (PP).

**Results:**

Data from 29 women in group 1, 25 women in group 2 and 33 women in group 3 were included for analysis. FLA, FLV, CRAE, CRVE, AVR and SveinO_2_ were altered in group 2 (p‐values between < 0.001 and 0.009). At TP 3 the differences between groups were most pronounced. In contrast, there were only minor differences between group 1 and 3. Changes in retinal parameters were independently associated with systemic blood pressure and BMI.

**Conclusions:**

The present analysis indicates that flicker‐induced retinal vasodilation, retinal vessel diameters and retinal oxygen saturation are altered in high‐risk pregnant women. Hence, these parameters are candidate biomarkers for pregnancy complications, a hypothesis that deserves further study.

## Introduction

The retinal vasculature is the only part of the human body that allows for the direct visualization of microvessels. Alterations in retinal vessels and retinal perfusion have been reported in ocular disease such as glaucoma (Kawasaki et al. 2013; Van Melkebeke et al. 2018), retinitis pigmentosa (Lang et al. 2019), systemic hypertension (Ding et al. 2014; Chua et al. 2019), hypercholesterolaemia (Kelly et al. 2011; Nagele et al. 2018) and in patients with diabetes, where changes in the ocular vasculature occur even before the clinical onset of diabetic retinopathy (Pemp et al. 2010; Sabanayagam et al. 2015; Fondi et al. 2017; Rosen et al. 2019).

It is known that obesity of the mother is an important risk factor for complications during pregnancy (Marchi et al. 2015; Catalano & Shankar 2017) such as the development of preeclampsia, which is associated with an increased risk of maternal and foetal morbidity and mortality.(Mol et al. 2016) The risk of preeclampsia doubles with each 5 to 7 kg/m^2^ increase of BMI in pregnant women (Verma & Shrimali 2012) and women morbidly obese have a 12 times higher chance for preeclampsia and higher risk for induced labour, emergency caesarean section, preterm and postpartum haemorrhage in comparison to underweight women (Bhattacharya et al. 2007). In addition, abundant studies exhibited that preeclampsia can cause up to 15% of preterm deliveries and 25% of small‐for‐gestational‐age (SGA; Zhang et al. 2001).

Endothelial dysfunction has been suggested as a biomarker for the risk of adverse pregnancy outcome (Boeldt & Bird 2017), but techniques to assess endothelial function *in vivo* such as plethysmography of the forearm circulation or flow‐mediated vasodilation of brachial artery are time consuming (Flammer et al. 2012). Therefore, several studies have followed the idea to study the retinal microvasculature as a potential biomarker in pregnancy (Lupton et al. 2013; Li et al. 2018).

The aim of the present study was to investigate whether there is a difference in retinal vascular parameters between high‐risk and low‐risk pregnant women over time during pregnancy. Flicker‐induced retinal vasodilatation, which has been found to correlate with flow‐mediated vasodilatation in the brachial artery in previous studies (Pemp et al. 2009b) was used to assess neurovascular coupling (Riva et al. 2005; Kur et al. 2012). We used retinal oximetry, a technique based on spectroscopic analysis of fundus photographs, to measure retinal arterial and venous oxygen saturation (SaO_2_, SvO_2_; Stefansson et al. 2019). In addition, retinal vessel diameters and arterio‐venous ratio (AVR) were assessed. Three groups of women were included: pregnant women with normal blood pressure and not overweight, pregnant women with hypertension and/or overweight and a group of age‐matched nonpregnant healthy women. Pregnant women were followed up longitudinally during pregnancy, while measurements in healthy controls were only performed once.

## Methods

### Subjects

The present study was performed according to the Good Clinical Practice guidelines and the Declaration of Helsinki. Prior to the start of the study, we obtained approval by the Ethics Committee of the Medical University of Vienna, Austria. We obtained written informed consent from all participating women prior to inclusion in the study.

A total of 115 women were included in three groups. In all participating subjects, a screening investigation was performed including medical history, smoking status (current, past), physical examination including 12‐lead electrocardiogram (ECG) and measurement of systemic haemodynamics, and an ophthalmic examination (best corrected visual acuity, slit‐lamp biomicroscopy, indirect funduscopy and measurement of IOP). Only women with normal ophthalmic findings as judged by the investigator were included in this study. All subjects had to be ≥18 years of age to be eligible for inclusion. Group 1 consisted of 38 pregnant women with normal blood pressure (systolic blood pressure (SBP) < 140 mmHg and diastolic blood pressure (DBP) < 90 mmHg) and normal BMI (≥18.5 and <25 kg/m^2^). Group 2 consisted of women with either systemic hypertension (SBP > 140 mmHg and/or DBP < 90 mmHg) and/or overweight (BMI > 25 kg/m^2^). Women with pre‐existing hypertension as well as women with acquired hypertension during pregnancy were included. In pregnant women, inclusion/exclusion criteria were evaluated between pregnancy weeks (wks) 20 and 28. Group 3 consisted of age‐matched nonpregnant women with blood pressure and BMI levels in the normal range as defined above for group 1. Diabetes mellitus and alcohol abuse were exclusion criteria in all groups.

Sample size calculation was based on the reproducibility of the main outcome variable (flicker response of retinal vessels) in our laboratory and a power of 0.8 (Told et al. 2014). The study was designed to detect a difference between groups 1 and 2 at a two‐sided 0.05 significance level, if the true difference between groups is ±25%. This was based on our previous results that the standard deviation of the main outcome variable is approximately 35% (unpublished data from our laboratory). Differences in the main outcome variable smaller than 25% were considered irrelevant. These assumptions resulted in a sample size of 30 women per group. To account for a 20% drop out rate, we increased the sample size to 36 per group.

### Study protocol

Pregnant women were recruited at the Department of Obstetrics and Gynecology, Medical University of Vienna, Austria. The nonpregnant control group (group 3) was recruited at the Department of Clinical Pharmacology, Medical University of Vienna, Austria. All retinal parameters were measured at the Department of Clinical Pharmacology.

Study visit 1 was scheduled between pregnancy wks 20 and 28 (2nd trimester, TP 2), study visit 2 was scheduled between wks 30 and 34 (3rd trimester, TP 3) and study visit 3 was scheduled postpartum, day 1–5 (PP). In nonpregnant women, examinations were performed only once. On all study days, the same schedule of measurements was applied for all subjects. The right eye was used as the study eye. For pupil dilation, one drop of tropicamide eye drops (Mydriaticum Agepha, Agepha, Vienna, Austria) was instilled into the study eye and after a 20‐min resting period, measurements were performed with the dynamic vessel analyser (DVA, IMEDOS GmbH, Jena, Germany). Blood pressure and pulse rate were obtained by an automated oscillometric device (Infinity Delta, Dräger, Vienna, Austria). Intraocular pressure (IOP) was measured using applanation tonometry. The BMI was taken at the first visit.

### Dynamic vessel analyser

The DVA was used for the measurement of vessel diameters and the assessment of flicker‐induced vasodilation. The DVA has been described in detail in a previous review paper (Garhofer et al. 2010). Briefly, the system consists of a fundus camera, a video recorder and a system for stimulation with diffuse luminance flicker. After a 1‐min baseline measurement, the retina is stimulated with flicker light for another minute. Baseline vessel diameters were determined by averaging values over the last 20 s before flicker stimulation. During flicker stimulation, vessel diameters were averaged over the last 20 s (Garhofer et al. 2010). Flicker‐induced retinal vasodilation was defined as the relative increase from baseline diameter values during flicker stimulation. This was calculated for retinal arteries (FLA) and retinal veins (FLV) separately.

#### Flicker stimulus

For flicker stimulation, the built‐in stimulation system of the DVA device was used. The flicker stimulus is generated by an electronic shutter system, which interrupts the background illumination of the fundus camera of the DVA device at a frequency of 12.5 Hz. This results in a square wave stimulation pattern with a frequency of 12.5 Hz, a modulation depth of 100% and a contrast ratio of 25:1.

For static vessel analysis, a fundus photograph was taken and the analysis was carried out as previously described (Nagel et al. 2007). Briefly, the Visualis system (IMEDOS GmbH, Jena, Germany) was used to acquire the monochromatic fundus images (535–561 nm, 30° imaging angle) and the photographs were analysed with a concentric ring around the optic nerve head using Vesselmap2 analysis software (IMEDOS GmbH, Jena, Germany). Calculation of the central retinal arterial equivalent (CRAE), central venous equivalent (CRVE) and the AVR was done according to the Parr‐Hubbard formula (Parr & Spears 1974; Hubbard et al. 1999).

The same device was used to measure retinal haemoglobin oxygen saturation (SO_2_) based on optical reflectometry using the commercially available software package (RVA, IMEDOS GmbH, Jena, Germany). Briefly, two simultaneously recorded 50° monochromatic fundus images are taken at 548 and 610 nm, respectively. Then the optical density ratio of the blood vessels in these two images was calculated and SO_2_ values were determined for retinal arteries (SartO_2_) and retinal veins (SveinO_2_), taking the vessel diameter into account. In a previous study, it was shown that SO_2_ values are dependent on vessel diameters (Hammer et al. 2008). The Imedos software incorporates the correction formulas as provided in this paper. In the present study, all retinal arteries and veins were measured at a distance of 1.0 to 1.5 disc diameters to the optic nerve head.

### Measurement of systemic blood pressure and pulse rate

Automated oscillometry was used to measure systolic, diastolic, mean arterial blood pressures and pulse rate (SBP, DBP, MAP, PR) on the upper arm (Infinity Delta, Dräger). Single measurements were performed at the screening visit and on each study day.

### Measurement of intraocular pressure

IOP was measured with a slit‐lamp mounted Goldmann applanation tonometer. Before measurements, one drop of oxybuprocainhydrochloride combined with sodium fluorescein (Fluoresceine‐Oxybuprocaine SDU Faure, OmniVision AG, Neuhausen, Switzerland) was instilled.

### Data analysis

Shapiro–Wilk test was used to test the outcome parameters for normal distribution. All retinal outcome parameters were corrected for smoking status. Data for FLA and FLV were adjusted for vessel size, as this has an important effect on the amount of flicker‐induced vasodilation (Sharifizad et al. 2016). A repeated measures ancova model was used to compare the time course of all outcome parameters between groups 1 and 2. Baseline values between groups 1, 2 and 3 were compared using a one‐way ancova model. For groups 1 and 2, first a linear and second a subsequent multiple linear regression model was applied analysing the effect of the following factors on flicker responses: MAP, BMI, PR and age. Factors significant in the linear regression model were used for the multiple regression approach. This analysis was done separately for TP2 and TP3. A p‐value < 0.05 was considered as the level of significance for all analyses, which were performed using spss (IBM SPSS Statistics, Version 22, Armonk, NY, USA).

## Results

**Fig. 1:**
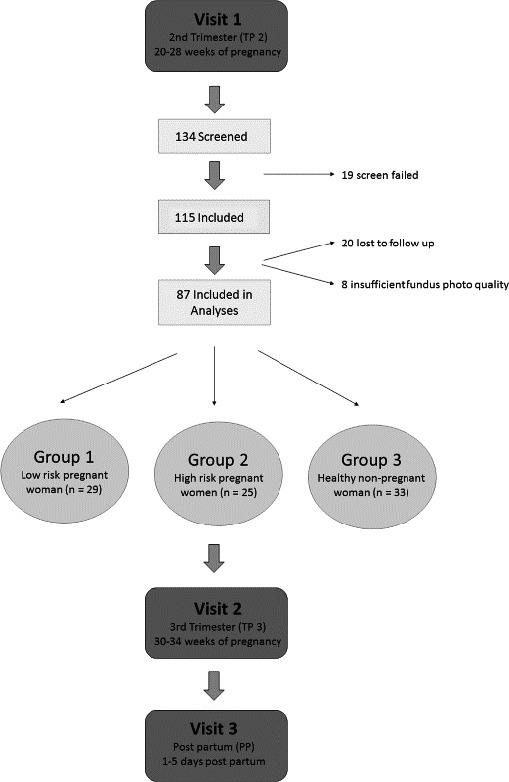
Study flow chart.

A total of 134 subjects were screened for eligibility, of which 19 were not included because the inclusion/exclusion criteria were not met. Out of the remaining 115 subjects that were eligible, 28 were not included for analysis because of loss to follow‐up (*n* = 20) or insufficient fundus photograph quality (*n* = 8). Hence, data from 29 women in group 1 (low‐risk pregnancy), 25 women in group 2 (high‐risk pregnancy) and 33 women in group 3 (healthy, non‐pregnant women) were analysed. A study flow chart is provided in Fig. 1. Out of the 25 subjects analysed in group 2, 17 had systemic hypertension and 23 were overweight.

All outcome parameters were normally distributed. There was no significant difference in age between the three groups (group 1: 28.1 ± 5.1 years, group 2: 29.2 ± 5.4 years, group 3: 27.8 ± 4.9 years; p = 0.196). The number of smokers differed significantly between the three included groups (p < 0.001). The proportion of current (group 1: *n* = 4 (14%), group 2: *n* = 9 (36%), group 3: *n* = 0 (0%)) as well as past smokers (group 1: *n* = 3 (10%), group 2: *n* = 6 (24%), group 3: *n* = 1 (3%)) was highest in group 2 followed by group 1. BMI was within the normal range and not different between groups 1 (22.7 ± 4.4 kg/m^2^) and groups 3 (21.8 ± 4.1 kg/m^2^), but significantly elevated in group 2 (29.9 ± 6.3 kg/m^2^; p < 0.001 versus group 1 and 3). The time course of MAP, PR and IOP is presented in Table 1. Both MAP and PR were significantly higher in group 2 as compared to the other groups at the second study visit (TP 3, p < 0.001). In group 1 MAP and PR did not change over time. By contrast, MAP increased at TP3 in group 2 (p = 0.002) and PR and tended to increase (p = 0.061). No differences in IOP were observed between groups.

**Table 1:** Body mass index (BMI), Mean arterial blood pressure (MAP), pulse rate (PR) and intraocular pressure (IOP) in the three patient groups for the time‐points TP 2 (2nd trimester, wks 20–28), TP 3 (3rd trimester, wks 30–34) and PP (1‐ to 5‐day postpartum)

	TP2	TP3	PP	p‐value
BMI (kg/m^2^)				
Group 1 (low‐risk pregnancy, *n* = 29)	22.7 ± 4.4			<0.001^##^
Group 2 (high‐risk pregnancy, *n* = 25)	29.9 ± 6.3			
Group 3 (nonpregnant healthy controls, *n* = 33)	21.8 ± 4.1			
MAP (mmHg)				
Group 1 (low‐risk pregnancy, *n* = 29)	90 ± 5	91 ± 5	89 ± 4	<0.001^#^
Group 2 (high‐risk pregnancy, *n* = 25)	110 ± 8	115 ± 9	108 ± 8	
Group 3 (nonpregnant healthy controls, *n* = 33)	87 ± 4			<0.001^##^
PR (beats/min)				
Group 1 (low‐risk pregnancy, *n* = 29)	62 ± 7	61 ± 7	60 ± 6	0.003^#^
Group 2 (high‐risk pregnancy, *n* = 25)	70 ± 9	72 ± 10	72 ± 9	
Group 3 (nonpregnant healthy controls, *n* = 33)	55 ± 7			<0.001^##^
IOP (mmHg)				
Group 1 (low‐risk pregnancy, *n* = 29)	15.4 ± 3.2	15.1 ± 2.9	15.3 ± 3.1	0.476^#^
Group 2 (high‐risk pregnancy, *n* = 25)	15.7 ± 3.5	15.8 ± 3.6	15.9 ± 3.3	
Group 3 (nonpregnant healthy controls, *n* = 33)	15.6 ± 3.0			0.736^##^

A repeated measures ancova model was used to compare Groups 1 and 2 (#), a 3‐way ancova model was used to compare Groups 1, 2 and 3 (##).

The results for flicker‐induced vasodilation are presented in Table 2. In group 2, FLA was lower than in groups 1 and 3 at the first study visit (TP2). At TP3, FLA in group 2 decreased further (p < 0.001), but returned towards values observed at TP2 at the postpartum visit (PP). The response of retinal veins to flicker stimulation followed a similar pattern. Again, FLV was lower in group 2 as compared to the other groups and decreased further at TP 3 (p < 0.001), returning to values near TP 2 at PP. In group 1, FLV decreased at TP 3 and returned to TP 2 values at PP (p = 0.021). Vessel diameter and oxygen saturation results are presented in Table 3. CRAE was not different between groups 1 and 3, but was significantly more narrow in group 2 (p < 0.001). In addition, CRAE showed a further reduction at TP 3 in group 2 (p = 0.006), again returning near to TP 2 values at PP. In retinal veins, the opposite effect was observed. CRVE was wider in group 2 than in groups 1 and 3 (p = 0.005). In groups 1 and 2 there was an increase in CRVE at TP 3, which was more pronounced in the women with hypertension and/or overweight (group 1: p = 0.032; group 2: p = 0.007). Due to arterial vasoconstriction and venous vasodilation, there was a pronounced AVR reduction in group 2 as compared to the other groups (p < 0.001), which got more pronounced at TP 3 (p = 0.003). SartO_2_ did not show any differences between groups. SveinO_2_ was higher in group 2 than in the other groups (p = 0.003). No time‐dependence of SveinO_2_ was observed.

**Table 2:** Flicker‐induced retinal vasodilation in retinal arteries (FLA) and veins (FLV) in the three patient groups for the time‐points TP 2 (2nd trimester, wks 20–28), TP 3 (3rd trimester, wks 30–34) and PP (1‐ to 5‐day postpartum)

	TP2	TP3	PP	p‐value
FLA (%)				
Group 1 (low‐risk pregnancy, *n* = 29)	5.7 ± 1.7	5.5 ± 1.3	5.8 ± 1.2	<0.001^#^
Group 2 (high‐risk pregnancy, *n* = 25)	3.1 ± 2.0	1.8 ± 1.5	3.4 ± 1.7	
Group 3 (nonpregnant healthy controls, *n* = 33)	6.1 ± 1.5			<0.001^##^
FLV (%)				
Group 1 (low‐risk pregnancy, *n* = 29)	6.3 ± 1.6	5.8 ± 1.5	6.2 ± 1.4	<0.001^#^
Group 2 (high‐risk pregnancy, *n* = 25)	4.0 ± 2.1	2.6 ± 1.4	3.8 ± 1.9	
Group 3 (nonpregnant healthy controls, *n* = 33)	6.5 ± 1.9			<0.001^##^

(#) indicates statistical significance between Groups 1 and 2 using a repeated measures ancova model. (##) indicates statistical significant difference comparing Groups 1, 2 and 3 using 3‐way ancova model.

**Table 3:** Retinal vessel diameters (central retinal artery equivalent (CRAE), central retinal vein equivalent (CRVE), AVR, oxygen saturation in retinal arteries (SartO_2_) and oxygen saturation in retinal veins (SveinO_2_) in the three patient groups for the time‐points TP 2 (2nd trimester, wks 20–28), TP 3 (3rd trimester, wks 30–34) and PP (1‐ to 5‐day postpartum)

	TP2	TP3	PP	p‐value
CRAE (*µ*m)				
Group 1 (low‐risk pregnancy, *n* = 29)	187 ± 19	185 ± 17	188 ± 20	<0.001^#^
Group 2 (high‐risk pregnancy, *n* = 25)	161 ± 20	152 ± 22	158 ± 19	
Group 3 (nonpregnant healthy controls, *n* = 33)	187 ± 17			<0.001^##^
CRVE (*µ*m)				
Group 1 (low‐risk pregnancy, *n* = 29)	234 ± 24	241 ± 26	232 ± 22	0.009^#^
Group 2 (high‐risk pregnancy, *n* = 25)	241 ± 27	250 ± 30	244 ± 28	
Group 3 (nonpregnant healthy controls, *n* = 33)	233 ± 23			0.022^##^
AVR				
Group 1 (low‐risk pregnancy, *n* = 29)	0.80 ± 0.11	0.77 ± 0.10	0.81 ± 0.11	< 0.001^#^
Group 2 (high‐risk pregnancy, *n* = 25)	0.67 ± 0.13	0.61 ± 0.12	0.65 ± 0.12	
Group 3 (nonpregnant healthy controls, *n* = 33)	0.81 ± 0.11			<0.001^##^
SartO_2_ (%)				
Group 1 (low‐risk pregnancy, *n* = 29)	94 ± 2	94 ± 3	93 ± 3	0.635^#^
Group 2 (high‐risk pregnancy, *n* = 25)	93 ± 2	93 ± 2	93 ± 2	
Group 3 (nonpregnant healthy controls, *n* = 33)	95 ± 2			0.331^##^
SveinO_2_ (%)				
Group 1 (low‐risk pregnancy, *n* = 29)	64 ± 2	65 ± 2	64 ± 2	0.008^#^
Group 2 (high‐risk pregnancy, *n* = 25)	68 ± 2	69 ± 2	68 ± 2	
Group 3 (nonpregnant healthy controls, *n* = 33)	64 ± 2			0.017^##^

(#) indicates statistical significance between Groups 1 and 2 using a repeated measures ancova model. (##) indicates statistical significant difference comparing Groups 1, 2 and 3 using 3‐way ancova model.

The results of linear regression analysis at TP 2 are shown in Table 4. None of the retinal variables were age‐dependent. BMI was statistically significant linked to all retinal parameters except SartO_2_ and SveinO_2_. The majority of retinal parameters except CRVE and SartO_2_ were associated with MAP. By contrast, all parameters except CRAE were not correlated to PR. BMI was another factor linked to retinal parameters and significant for all factors except SartO_2_ and SveinO_2_.

**Table 4:** Results of linear regression analysis in pregnant women at TP 2 (2nd trimester, wks 20–28, group 1 + group 2: *n* = 54)

	MAP (mmHg)	PR (beats/min)	BMI (kg/m^2^)	Age (years)
TP2				
FLA (%)	***r* = ‐ 0.41**	*r* = −0.11	***r* = −0.36**	*r* = 0.02
	**p = 0.002**	p = 0.43	**p = 0.007**	p = 0.89
FLV (%)	***r* = −0.31**	*r* = −0.04	***r* = −0.54**	*r* = −0.13
	**p = 0.022**	p = 0.77	**p < 0.001**	p = 0.35
CRAE (*µ*m)	***r* = −0.50**	***r* = −0.27**	***r* = −0.40**	*r* = −0.23
	**p < 0.001**	**p = 0.048**	**p = 0.003**	p = 0.09
CRVE (*µ*m)	*r* = 0.11	*r* = 0.07	***r* = 0.38**	*r* = 0.15
	p = 0.43	p = 0.61	**p = 0.005**	p = 0.28
AVR	***r* = −0.55**	*r* = −0.12	***r* = −0.47**	*r* = −0.02
	**p < 0.001**	p = 0.38	**p < 0.001**	p = 0.89
SartO_2_ (%)	*r* = −0.09	*r* = 0.13	*r* = −0.01	*r* = −0.10
	p = 0.52	p = 0.35	p = 0.94	p = 0.47
SveinO_2_ (%)	***r* = 0.41**	*r* = 0.18	*r* = 0.22	*r* = 0.17
	**p = 0.002**	p = 0.19	p = 0.10	p = 0.18

A p‐value < 0.05 was considered as the level of significance (bold values).

The results of the multiple regression model are presented in Table 5. MAP remained significantly associated with FLA, FLV, CRAE, AVR and SveinO_2_. BMI was a determinant of retinal venous parameters (FLV, CRVE) except SveinO_2_, but also of FLA, CRAE and AVR.

**Table 5:** Results of the multiple regression model at TP 2 (2^nd^ trimester, wks 20‐28, group 1 + group 2: *n* = 54)

	Multivariate analysis	
	Coefficients (95% CI)	p‐value
FLA (%)		
MAP (mmHg)	−1.98 (−1.64,−2.41)	0.005
BMI (kg/m^2^)	−2.23 (−1.41,−3.07)	0.012
FLV (%)		
MAP (mmHg)	−1.63 (−1.21,−2.11)	0.038
BMI (kg/m^2^)	−2.46 (−1.67,−3.34)	0.009
CRAE (*µ*m)		
MAP (mmHg)	−1.94 (−1.55,−2.48)	<0.001
PR (beats/min)	−1.41 (−0.76.−2.17)	0.365
BMI (kg/m^2^)	−2.17 (−1.34,−3.22)	0.040
CRVE (*µ*m)		
BMI (kg/m^2^)	2.24 (1.56,3.04)	0.005
AVR		
MAP (mmHg)	−2.08 (−1.54,−2,77)	<0.001
BMI (kg/m^2^)	−2.87 (−2.02,−4.01)	<0.001
SveinO_2_ (%)		
MAP (mmHg)	2.17 (1.74, 2.80)	0.002

The results of linear and multiple regression analysis at TP 3 are shown in Tables 6 and 7, respectively. The results were generally comparable to those obtained at TP 2, but correlations were stronger. In the linear regression analysis, a significant association was found between CRVE and MAP, FLV and PR, as well as SveinO_2_ and BMI. These correlations were not found at TP 2. By contrast, the correlation between CRAE and PR was not significant at TP 3.

**Table 6:** Results of linear regression analysis in pregnant women at TP 3 (3^rd^ trimester, wks 30‐34, group 1 + group 2: *n* = 54)

	MAP (mmHg)	PR (beats/min)	BMI (kg/m^2^)	Age (years)
TP3				
FLA (%)	***r* = −0.52**	*r* = −0.17	***r* = −0.46**	*r* = 0.13
	**p < 0.001**	p = 0.22	**p < 0.001**	p = 0.35
FLV (%)	***r* = −0.30**	***r* = −0.27**	***r* = −0.66**	*r* = −0.07
	**p = 0.028**	**p = 0.048**	**p < 0.001**	p = 0.61
CRAE (*µ*m)	***r* = −0.64**	*r* = −0.22	***r* =** −**0.45**	*r* = −0.07
	**p < 0.001**	p = 0.11	**p < 0.001**	p = 0.62
CRVE (*µ*m)	***r* = 0.29**	*r* = 0.20	***r* = 0.52**	*r* = 0.15
	**p = 0.033**	p = 0.15	**p < 0.001**	p = 0.28
AVR	***r* = −0.67**	*r* = 0.01	***r* = −0.56**	*r* = −0.06
	**p < 0.001**	p = 0.94	**p < 0.001**	p = 0.67
SartO_2_ (%)	*r* = −0.14	*r* = −0.10	*r* = −0.01	*r* = −0.10
	p = 0.31	p = 0.47	p = 0.94	p = 0.47
SveinO_2_ (%)	***r* = 0.37**	*r* = 0.24	***r* = 0.31**	*r* = 0.09
	**p = 0.006**	p = 0.08	**p = 0.022**	p = 0.52

A p‐value < 0.05 was considered as the level of significance (bold values).

**Table 7:** Results of the multiple regression model at TP 3 (3^rd^ trimester, wks 30‐34, group 1 + group 2: *n* = 54)

	Multivariate analysis	
	Coefficients (95%CI)	p‐value
FLA (%)		
MAP (mmHg)	−2.13 (−1.77,−2.57)	<0.001
BMI (kg/m^2^)	−2.27 (−1.59,−2.85)	0.005
FLV (%)		
MAP (mmHg)	−1.84 (−1.33,−2.25)	0.023
PR (beats/min)	−1.45 (−0.91,−2.00)	0.176
BMI (kg/m^2^)	−2.53 (−1.75,−3.41)	0.008
CRAE (*µ*m)		
MAP (mmHg)	−2.24 (−1.77,−2.66)	<0.001
BMI (kg/m^2^)	−2.09 (−1.36,−2.84)	0.031
CRVE (*µ*m)		
MAP (mmHg)	2.09 (1.66,2.60)	<0.001
BMI (kg/m^2^)	2.40 (1.81,3.41)	<0.001
AVR		
MAP (mmHg)	−2.25 (−1.77,−2,92)	<0.001
BMI (kg/m^2^)	−2.91 (−2.11,−3.86)	<0.001
SveinO_2_ (%)		
MAP (mmHg)	2.26 (1.80, 2.96)	<0.001
BMI (kg/m^2^)	1.42 (0.91, 1.93)	0.088

## Discussion

Endothelial dysfunction has been proposed as a biomarker for preterm birth and small‐for‐gestational‐age deliveries (Chen & Scholl 2014; Amaral et al. 2017). A recent study was, however, not able to establish endothelial dysfunction in nulliparous women measured 3 years before pregnancy as a risk factor for preterm birth and/or small‐for‐gestational‐age deliveries (Lane‐Cordova et al. 2018). The present study looked into the potential use of retinal vascular parameters as biomarkers for risk pregnancies. The main finding of our study is that abnormalities in retinal vascular factors were observed in a group of pregnant women with systemic hypertension and/or overweight. Flicker‐induced retinal vasodilation, retinal vessel diameters as well as retinal oxygen saturation were abnormal in this group of women with risk factors for preterm birth and/or small‐for‐gestational‐age deliveries.

The idea of using retinal vascular parameters for risk assessment in pregnancy is not new. A wide variety of studies have looked into the association between retinal vessel diameters and pregnancy risk factors (Li et al. 2012; Lupton et al. 2013; Li et al. 2018). A recent study showed that increased blood pressure during pregnancy was associated with abnormal retinal vessel diameters, but independent of postpartum cardiovascular risks (Sim et al. 2019). The former result is in keeping with our findings that abnormalities in retinal vessel diameters are associated with systemic blood pressure. In addition, we have established that retinal venous dilatation is associated with BMI. The link between these parameters may be mediated via inflammation as previously shown for various diseases (Wong et al. 2006; Daien et al. 2013; Jabs et al. 2019). In addition, CRAE was significantly reduced in high‐risk pregnant women compared to low‐risk pregnant women and healthy nonpregnant controls. This is probably caused by the accompanying increase in MAP, since it is well established and has also been found in the present study that higher mean arterial pressure comes with retinal arteriolar narrowing (Chew et al. 2012).

Our results on altered flicker‐induced retinal vasodilation in risk pregnant women are well compatible with previous studies in preeclampsia (Bruckmann et al. 2015). The mechanisms underlying flicker‐induced retinal vasodilation are, however, not yet fully elucidated (Kur et al. 2012; Newman 2013; Nippert et al. 2018). It has previously been shown that inhibition of nitric oxide synthase blunts both FLA and FLV suggesting a role of endogenous nitric oxide in mediating or modulating flicker‐induced vasodilatation (Buerk et al. 1996; Dorner et al. 2003). This supports our hypothesis that FLA and FLV are potential biomarkers for pregnancy complications, because gasotransmitters such as nitric oxide, carbon monoxide and hydrogen sulphide have been implicated in preeclampsia and preterm birth and it seems that nitric oxide production, nitric oxide synthase activation as well as expression is downregulated in preeclampsia (Rengarajan et al. 2020). In patients with type 1 diabetes, flicker‐induced vasodilatation was reduced while the vascular response to exogenous nitric oxide was not altered.(Pemp et al. 2009a; Pemp et al. 2009b) Some authors have proposed that the response of the retinal vasculature is a measure of endothelial dysfunction, but this hypothesis remains largely unproven (Lim et al. 2013). In the present study, we identified blood pressure and BMI as determinants of flicker‐induced retinal vasodilation. Since correlations were stronger at TP 3 compared to TP 2, we also conclude that these parameters determine the further reduction at week 30–34 as seen in group 2. Previous studies have linked FLA and FLV to diabetes (Garhofer et al. 2004; Sorensen et al. 2016), systemic hypertension (Nagel et al. 2004; Machalinska et al. 2018), cardiovascular disease (Heitmar et al. 2017; Heitmar et al. 2018), hypercholesterolaemia (Sharifizad et al. 2016; Nagele et al. 2018), obesity (Kotliar et al. 2011) and even to asymptomatic individuals with a positive family history of cardiovascular disease (Seshadri et al. 2018).

Whereas SartO_2_ was not different in pregnant women versus healthy controls, group 2 showed increased SveinO_2_. Alterations in retinal oxygen saturation seem to be linked to many ocular and systemic diseases such as diabetes, glaucoma, retinopathy of prematurity as well as respiratory, cardiovascular and neurological diseases (Stefansson et al. 2019). Increased SveinO_2_ at normal SartO_2_ indicates reduced arterio‐venous oxygen difference and reduced retinal oxygen extraction (Palkovits et al. 2014a, 2014b; Felder et al. 2015; Werkmeister et al. 2015; Fondi et al. 2017; Aref et al. 2019; Bata et al. 2019; Blair et al. 2019), although this requires experimental verification. The present study established BMI as a determinant of SveinO_2_ in pregnant women, a result that warrants further investigations in other diseases.

A strength of the present study is the assessment of several retinal vascular parameters that all can be measured with the same device within 10 min. Other authors have proposed other approaches such as measurement of vessel density using optical coherence tomography angiography (Chanwimol et al. 2019; Kiziltunc et al. 2020), retinal blood flow using laser Doppler velocimetry (Chen et al. 1994) or optic nerve head blood flow as assessed using laser speckle flowgraphy (Sato et al. 2017). However, we do not know how these approaches compare to flicker‐induced retinal vasodilation, retinal vessel diameters or retinal oxygen saturation as measured in the present study. Another strength relates to the inclusion of a nonpregnant control group that showed that pregnancy in the low‐risk group was not associated with major changes in the retinal vasculature. Finally, we used a longitudinal approach that showed that retinal vascular abnormalities in the high‐risk group were most pronounced at TP3. Whether the change in retinal vasculature over time during pregnancy may in itself be a potential biomarker for preterm birth and/or small‐for‐gestational‐age deliveries requires further study.

A limitation of the study is the relatively low sample size, which was based on the sample size calculation for the main outcome variable (flicker‐induced vasodilatation). As such we were also unable to sufficiently correlate the parameters to preterm birth and/or small‐for‐gestational‐age delivery. The present study was not designed to answer this question and a larger scale study needs to be planned to support this hypothesis. Moreover, we did not include women with preeclampsia, although endothelial dysfunction has been clearly linked to this condition (Possomato‐Vieira & Khalil 2016). In the group of pregnant women, we did not obtain measurements before pregnancy or earlier in pregnancy due to logistical reasons. This also means that our BMIs were taken in groups 1 and 2 when women were pregnant already. By including a healthy control group we did, however, show that values obtained in group 1 at TP 2 were not different from normal. Different authors have used different protocols for flicker‐induced vasodilation using the DVA (Garhofer et al. 2010; Sharifizad et al. 2016). We chose a protocol using one flicker period only in order to limit the discomfort for the subjects under study. Finally, we did not include a long‐term follow‐up to investigate whether the retinal parameters are associated with the incidence of cardiovascular disease.

In conclusion, the present study showed that retinal microvascular factors as assessed using the DVA are associated with systemic hypertension and increased BMI in pregnant women. Flicker‐induced retinal vasodilation and retinal oxygen saturation are therefore potential novel biomarkers for pregnancy complications. Further studies are required to link these retinal vascular parameters to preeclampsia as well as preterm birth and/or small‐for‐gestational‐age deliveries.

## References

[aos14696-bib-0001] Amaral LM , Wallace K , Owens M & LaMarca B (2017): Pathophysiology and current clinical management of preeclampsia. Curr Hypertens Rep 19: 61. 28689331 10.1007/s11906-017-0757-7 PMC5916784

[aos14696-bib-0002] Aref AA , Maleki S , Tan O , Huang D , Varma R & Shahidi M (2019): Relating glaucomatous visual field loss to retinal oxygen delivery and metabolism. Acta Ophthalmol 97: e968–e972. 31016869 10.1111/aos.14120

[aos14696-bib-0003] Bata AM , Fondi K , Szegedi S et al. (2019): Age‐related decline of retinal oxygen extraction in healthy subjects. Invest Ophthalmol Vis Sci 60: 3162–3169. 31335953 10.1167/iovs.18-26234

[aos14696-bib-0004] Bhattacharya S , Campbell DM , Liston WA & Bhattacharya S (2007): Effect of Body Mass Index on pregnancy outcomes in nulliparous women delivering singleton babies. BMC Public Health 7: 168. 17650297 10.1186/1471-2458-7-168 PMC1940246

[aos14696-bib-0005] Blair NP , Tan MR , Felder AE & Shahidi M (2019): Retinal oxygen delivery, metabolism and extraction fraction and retinal thickness immediately following an interval of ophthalmic vessel occlusion in rats. Sci Rep 9: 8092. 31147557 10.1038/s41598-019-44250-y PMC6542852

[aos14696-bib-0006] Boeldt DS & Bird IM (2017): Vascular adaptation in pregnancy and endothelial dysfunction in preeclampsia. J Endocrinol 232: R27–R44. 27729465 10.1530/JOE-16-0340 PMC5115955

[aos14696-bib-0007] Bruckmann A , Seeliger C , Lehmann T , Schleussner E & Schlembach D (2015): Altered retinal flicker response indicates microvascular dysfunction in women with preeclampsia. Hypertension 66: 900–905. 26283041 10.1161/HYPERTENSIONAHA.115.05734

[aos14696-bib-0008] Buerk DG , Riva CE & Cranstoun SD (1996): Nitric oxide has a vasodilatory role in cat optic nerve head during flicker stimuli. Microvasc Res 52: 13–26. 8812749 10.1006/mvre.1996.0040

[aos14696-bib-0009] Catalano PM & Shankar K (2017): Obesity and pregnancy: mechanisms of short term and long term adverse consequences for mother and child. BMJ 356: j1. 28179267 10.1136/bmj.j1 PMC6888512

[aos14696-bib-0010] Chanwimol K , Balasubramanian S , Nassisi M , Gaw SL , Janzen C , Sarraf D , Sadda SR & Tsui I (2019): Retinal vascular changes during pregnancy detected with optical coherence tomography angiography. Invest Ophthalmol Vis Sci 60: 2726–2732. 31247113 10.1167/iovs.19-26956

[aos14696-bib-0011] Chen HC , Newsom RS , Patel V , Cassar J , Mather H & Kohner EM (1994): Retinal blood flow changes during pregnancy in women with diabetes. Invest Ophthalmol Vis Sci 35: 3199–3208. 8045714

[aos14696-bib-0012] Chen X & Scholl TO (2014): Maternal biomarkers of endothelial dysfunction and preterm delivery. PLoS One 9: e85716. 24465662 10.1371/journal.pone.0085716 PMC3899071

[aos14696-bib-0013] Chew SK , Xie J & Wang JJ (2012): Retinal arteriolar diameter and the prevalence and incidence of hypertension: a systematic review and meta‐analysis of their association. Curr Hypertens Rep 14: 144–151. 22322543 10.1007/s11906-012-0252-0

[aos14696-bib-0014] Chua J , Chin CWL , Hong J , Chee ML , Le TT , Ting DSW , Wong TY & Schmetterer L (2019): Impact of hypertension on retinal capillary microvasculature using optical coherence tomographic angiography. J Hypertens 37: 572–580. 30113530 10.1097/HJH.0000000000001916 PMC6365272

[aos14696-bib-0015] Daien V , Carriere I , Kawasaki R , Cristol JP , Villain M , Fesler P , Ritchie K & Delcourt C (2013): Retinal vascular caliber is associated with cardiovascular biomarkers of oxidative stress and inflammation: the POLA study. PLoS One 8: e71089. 23923054 10.1371/journal.pone.0071089 PMC3724806

[aos14696-bib-0016] Ding J , Wai KL , McGeechan K et al. (2014): Retinal vascular caliber and the development of hypertension: a meta‐analysis of individual participant data. J Hypertens 32: 207–215. 24322199 10.1097/HJH.0b013e32836586f4 PMC4120649

[aos14696-bib-0017] Dorner GT , Garhofer G , Kiss B , Polska E , Polak K , Riva CE & Schmetterer L (2003): Nitric oxide regulates retinal vascular tone in humans. Am J Physiol Heart Circ Physiol 285: H631–H636. 12750062 10.1152/ajpheart.00111.2003

[aos14696-bib-0018] Felder AE , Wanek J , Blair NP & Shahidi M (2015): Inner retinal oxygen extraction fraction in response to light flicker stimulation in humans. Invest Ophthalmol Vis Sci 56: 6633–6637. 26469748 10.1167/iovs.15-17321 PMC4611954

[aos14696-bib-0019] Flammer AJ , Anderson T , Celermajer DS et al. (2012): The assessment of endothelial function: from research into clinical practice. Circulation 126: 753–767. 22869857 10.1161/CIRCULATIONAHA.112.093245 PMC3427943

[aos14696-bib-0020] Fondi K , Wozniak PA , Howorka K et al. (2017): Retinal oxygen extraction in individuals with type 1 diabetes with no or mild diabetic retinopathy. Diabetologia 60: 1534–1540. 28547132 10.1007/s00125-017-4309-0 PMC5491565

[aos14696-bib-0021] Garhofer G , Bek T , Boehm AG et al. (2010): Use of the retinal vessel analyzer in ocular blood flow research. Acta Ophthalmol 88: 717–722. 19681764 10.1111/j.1755-3768.2009.01587.x

[aos14696-bib-0022] Garhofer G , Zawinka C , Resch H , Kothy P , Schmetterer L & Dorner GT (2004): Reduced response of retinal vessel diameters to flicker stimulation in patients with diabetes. Br J Ophthalmol 88: 887–891. 15205231 10.1136/bjo.2003.033548 PMC1772243

[aos14696-bib-0023] Hammer M , Vilser W , Riemer T & Schweitzer D (2008): Retinal vessel oximetry‐calibration, compensation for vessel diameter and fundus pigmentation, and reproducibility. J Biomed Opt 13: 054015. 19021395 10.1117/1.2976032

[aos14696-bib-0024] Heitmar R , Lip GYH , Ryder RE & Blann AD (2017): Retinal vessel diameters and reactivity in diabetes mellitus and/or cardiovascular disease. Cardiovasc Diabetol 16: 56. 28446234 10.1186/s12933-017-0534-6 PMC5406879

[aos14696-bib-0025] Heitmar R , Nicholl P , Lee B , Lau YC & Lip G (2018): The relationship of systemic markers of haemostasis with retinal blood vessel responses in cardiovascular disease and/or diabetes. Br J Biomed Sci 75: 116–121. 29521170 10.1080/09674845.2017.1420130

[aos14696-bib-0026] Hubbard LD , Brothers RJ , King WN et al. (1999): Methods for evaluation of retinal microvascular abnormalities associated with hypertension/sclerosis in the Atherosclerosis Risk in Communities Study. Ophthalmology 106: 2269–2280. 10599656 10.1016/s0161-6420(99)90525-0

[aos14696-bib-0027] Jabs DA , Van Natta ML , Trang G et al. (2019): Association of systemic inflammation with retinal vascular caliber in patients with AIDS. Invest Ophthalmol Vis Sci 60: 2218–2225. 31108552 10.1167/iovs.18-26070 PMC6528842

[aos14696-bib-0028] Kawasaki R , Wang JJ , Rochtchina E , Lee AJ , Wong TY & Mitchell P (2013): Retinal vessel caliber is associated with the 10‐year incidence of glaucoma: the Blue Mountains Eye Study. Ophthalmology 120: 84–90. 23062656 10.1016/j.ophtha.2012.07.007

[aos14696-bib-0029] Kelly ER , Plat J , Mensink RP & Berendschot TT (2011): Effects of long term plant sterol and ‐stanol consumption on the retinal vasculature: a randomized controlled trial in statin users. Atherosclerosis 214: 225–230. 21122856 10.1016/j.atherosclerosis.2010.10.038

[aos14696-bib-0030] Kiziltunc PB , Varli B , Buyuktepe TC & Atilla H (2020): Ocular vascular changes during pregnancy: an optical coherence tomography angiography study. Graefes Arch Clin Exp Ophthalmol 258: 395–401. 31754828 10.1007/s00417-019-04541-6

[aos14696-bib-0031] Kotliar KE , Lanzl IM , Schmidt‐Trucksass A , Sitnikova D , Ali M , Blume K , Halle M & Hanssen H (2011): Dynamic retinal vessel response to flicker in obesity: a methodological approach. Microvasc Res 81: 123–128. 21094174 10.1016/j.mvr.2010.11.007

[aos14696-bib-0032] Kur J , Newman EA & Chan‐Ling T (2012): Cellular and physiological mechanisms underlying blood flow regulation in the retina and choroid in health and disease. Prog Retin Eye Res 31: 377–406. 22580107 10.1016/j.preteyeres.2012.04.004 PMC3418965

[aos14696-bib-0033] Lane‐Cordova AD , Gunderson EP , Carnethon MR et al. (2018): Pre‐pregnancy endothelial dysfunction and birth outcomes: The Coronary Artery Risk Development in Young Adults (CARDIA) Study. Hypertens Res 41: 282–289. 29449706 10.1038/s41440-018-0017-5 PMC6311125

[aos14696-bib-0034] Lang M , Harris A , Ciulla TA , Siesky B , Patel P , Belamkar A , Mathew S & Verticchio Vercellin AC (2019): Vascular dysfunction in retinitis pigmentosa. Acta Ophthalmol 97: 660–664. 31099494 10.1111/aos.14138

[aos14696-bib-0035] Li L‐J , Cheung CY‐L , Ikram MK et al. (2012): Blood pressure and retinal microvascular characteristics during pregnancy: Growing Up in Singapore Towards Healthy Outcomes (GUSTO) study. Hypertension 60: 223–230. 22615113 10.1161/HYPERTENSIONAHA.112.195404

[aos14696-bib-0036] Li L‐J , Tan KH , Aris IM et al. (2018): Retinal vasculature and 5‐year metabolic syndrome among women with gestational diabetes mellitus. Metabolism 83: 216–224. 29051041 10.1016/j.metabol.2017.10.004

[aos14696-bib-0037] Lim M , Sasongko MB , Ikram MK , Lamoureux E , Wang JJ , Wong TY & Cheung CY (2013): Systemic associations of dynamic retinal vessel analysis: a review of current literature. Microcirculation 20: 257–268. 23151190 10.1111/micc.12026

[aos14696-bib-0038] Lupton SJ , Chiu CL , Hodgson LAB et al. (2013): Temporal changes in retinal microvascular caliber and blood pressure during pregnancy. Hypertension 61: 880–885. 23399715 10.1161/HYPERTENSIONAHA.111.00698

[aos14696-bib-0039] Machalinska A , Pius‐Sadowska E , Babiak K , Salacka A , Safranow K , Kawa MP & Machalinski B (2018): Correlation between flicker‐induced retinal vessel vasodilatation and plasma biomarkers of endothelial dysfunction in hypertensive patients. Curr Eye Res 43: 128–134. 29135307 10.1080/02713683.2017.1358372

[aos14696-bib-0040] Marchi J , Berg M , Dencker A , Olander EK & Begley C (2015): Risks associated with obesity in pregnancy, for the mother and baby: a systematic review of reviews. Obes Rev 16: 621–638. 26016557 10.1111/obr.12288

[aos14696-bib-0041] Mol BWJ , Roberts CT , Thangaratinam S , Magee LA , de Groot CJM & Hofmeyr GJ (2016): Pre‐eclampsia. Lancet 387: 999–1011. 26342729 10.1016/S0140-6736(15)00070-7

[aos14696-bib-0042] Nagel E , Vilser W , Fink A & Riemer T (2007): Static vessel analysis in nonmydriatic and mydriatic images. Klin Monbl Augenheilkd 224: 411–416. 17516371 10.1055/s-2007-963093

[aos14696-bib-0043] Nagel E , Vilser W & Lanzl I (2004): Age, blood pressure, and vessel diameter as factors influencing the arterial retinal flicker response. Invest Ophthalmol Vis Sci 45: 1486–1492. 15111606 10.1167/iovs.03-0667

[aos14696-bib-0044] Nagele MP , Barthelmes J , Ludovici V , Cantatore S , Frank M , Ruschitzka F , Flammer AJ & Sudano I (2018): Retinal microvascular dysfunction in hypercholesterolemia. J Clin Lipidol 12: 1523–1531.e1522. 30219640 10.1016/j.jacl.2018.07.015

[aos14696-bib-0045] Newman EA (2013): Functional hyperemia and mechanisms of neurovascular coupling in the retinal vasculature. J Cereb Blood Flow Metab 33: 1685–1695. 23963372 10.1038/jcbfm.2013.145 PMC3824187

[aos14696-bib-0046] Nippert AR , Biesecker KR & Newman EA (2018): Mechanisms mediating functional hyperemia in the brain. Neuroscientist 24: 73–83. 28403673 10.1177/1073858417703033 PMC5757525

[aos14696-bib-0047] Palkovits S , Lasta M , Told R et al. (2014a): Retinal oxygen metabolism during normoxia and hyperoxia in healthy subjects. Invest Ophthalmol Vis Sci 55: 4707–4713. 25015353 10.1167/iovs.14-14593

[aos14696-bib-0048] Palkovits S , Told R , Schmidl D et al. (2014b): Regulation of retinal oxygen metabolism in humans during graded hypoxia. Am J Physiol Heart Circ Physiol 307: H1412–H1418. 25217648 10.1152/ajpheart.00479.2014

[aos14696-bib-0049] Parr JC & Spears GF (1974): Mathematic relationships between the width of a retinal artery and the widths of its branches. Am J Ophthalmol 77: 478–483. 4819452 10.1016/0002-9394(74)90458-9

[aos14696-bib-0050] Pemp B , Garhofer G , Weigert G , Karl K , Resch H , Wolzt M & Schmetterer L (2009a): Reduced retinal vessel response to flicker stimulation but not to exogenous nitric oxide in type 1 diabetes. Invest Ophthalmol Vis Sci 50: 4029–4032. 19369238 10.1167/iovs.08-3260

[aos14696-bib-0051] Pemp B , Polska E , Garhofer G , Bayerle‐Eder M , Kautzky‐Willer A & Schmetterer L (2010): Retinal blood flow in type 1 diabetic patients with no or mild diabetic retinopathy during euglycemic clamp. Diabetes Care 33: 2038–2042. 20585003 10.2337/dc10-0502 PMC2928359

[aos14696-bib-0052] Pemp B , Weigert G , Karl K , Petzl U , Wolzt M , Schmetterer L & Garhofer G (2009b): Correlation of flicker‐induced and flow‐mediated vasodilatation in patients with endothelial dysfunction and healthy volunteers. Diabetes Care 32: 1536–1541. 19478197 10.2337/dc08-2130 PMC2713642

[aos14696-bib-0053] Possomato‐Vieira JS & Khalil RA (2016): Mechanisms of endothelial dysfunction in hypertensive pregnancy and preeclampsia. Adv Pharmacol 77: 361–431. 27451103 10.1016/bs.apha.2016.04.008 PMC4965238

[aos14696-bib-0054] Rengarajan A , Mauro AK & Boeldt DS (2020): Maternal disease and gasotransmitters. Nitric Oxide 96: 1–12. 31911124 10.1016/j.niox.2020.01.001

[aos14696-bib-0055] Riva CE , Logean E & Falsini B (2005): Visually evoked hemodynamical response and assessment of neurovascular coupling in the optic nerve and retina. Prog Retin Eye Res 24: 183–215. 15610973 10.1016/j.preteyeres.2004.07.002

[aos14696-bib-0056] Rosen RB , Andrade Romo JS , Krawitz BD et al. (2019): Earliest evidence of preclinical diabetic retinopathy revealed using optical coherence tomography angiography perfused capillary density. Am J Ophthalmol 203: 103–115. 30689991 10.1016/j.ajo.2019.01.012 PMC6612596

[aos14696-bib-0057] Sabanayagam C , Lye WK , Klein R et al. (2015): Retinal microvascular calibre and risk of diabetes mellitus: a systematic review and participant‐level meta‐analysis. Diabetologia 58: 2476–2485. 26232097 10.1007/s00125-015-3717-2 PMC4751991

[aos14696-bib-0058] Sato T , Sugawara J , Aizawa N et al. (2017): Longitudinal changes of ocular blood flow using laser speckle flowgraphy during normal pregnancy. PLoS One 12: e0173127. 28257508 10.1371/journal.pone.0173127 PMC5336228

[aos14696-bib-0059] Seshadri S , Karimzad SE , Shokr H & Gherghel D (2018): Retinal vascular function in asymptomatic individuals with a positive family history of cardiovascular disease. Acta Ophthalmol 96: e956–e962. 30198216 10.1111/aos.13783

[aos14696-bib-0060] Sharifizad M , Witkowska KJ , Aschinger GC et al. (2016): Factors determining flicker‐induced retinal vasodilation in healthy subjects. Invest Ophthalmol Vis Sci 57: 3306–3312. 27333185 10.1167/iovs.16-19261

[aos14696-bib-0061] Sim R , Aris I , Chong YS , Wong TY & Li LJ (2019): Association between antenatal blood pressure and 5‐year postpartum retinal arteriolar structural and functional changes. BMJ Open Ophthalmol 4: e000355. 10.1136/bmjophth-2019-000355 PMC6936440 31909192

[aos14696-bib-0062] Sörensen BM , Houben AJHM , Berendschot TTJM et al. (2016): Prediabetes and Type 2 diabetes are associated with generalized microvascular dysfunction: the Maastricht study. Circulation 134: 1339–1352. 27678264 10.1161/CIRCULATIONAHA.116.023446

[aos14696-bib-0063] Stefansson E , Olafsdottir OB , Eliasdottir TS et al. (2019): Retinal oximetry: metabolic imaging for diseases of the retina and brain. Prog Retin Eye Res 70: 1–22. 30999027 10.1016/j.preteyeres.2019.04.001

[aos14696-bib-0064] Told R , Palkovits S , Boltz A et al. (2014): Flicker‐induced retinal vasodilatation is not dependent on complement factor H polymorphism in healthy young subjects. Acta Ophthalmol 92: e540–e545. 24863099 10.1111/aos.12433 PMC4225479

[aos14696-bib-0065] Van Melkebeke L , Barbosa‐Breda J , Huygens M & Stalmans I (2018): Optical coherence tomography angiography in glaucoma: a review. Ophthalmic Res 60: 139–151. 29794471 10.1159/000488495

[aos14696-bib-0066] Verma A & Shrimali L (2012): Maternal body mass index and pregnancy outcome. J Clin Diagn Res 6: 1531–1533. 23285448 10.7860/JCDR/2012/4508.2551 PMC3527788

[aos14696-bib-0067] Werkmeister RM , Schmidl D , Aschinger G et al. (2015): Retinal oxygen extraction in humans. Sci Rep 5: 15763. 26503332 10.1038/srep15763 PMC4621499

[aos14696-bib-0068] Wong TY , Islam FM , Klein R , Klein BE , Cotch MF , Castro C , Sharrett AR & Shahar E (2006): Retinal vascular caliber, cardiovascular risk factors, and inflammation: the multi‐ethnic study of atherosclerosis (MESA). Invest Ophthalmol Vis Sci 47: 2341–2350. 16723443 10.1167/iovs.05-1539 PMC2258139

[aos14696-bib-0069] Zhang J , Klebanoff MA & Roberts JM (2001): Prediction of adverse outcomes by common definitions of hypertension in pregnancy. Obstet Gynecol 97: 261–267. 11165592 10.1016/s0029-7844(00)01125-x

